# Characterization of the Highly Efficient Acid-Stable Xylanase and β-Xylosidase System from the Fungus *Byssochlamys spectabilis* ATHUM 8891 (*Paecilomyces variotii* ATHUM 8891)

**DOI:** 10.3390/jof7060430

**Published:** 2021-05-29

**Authors:** Anastasia P. Galanopoulou, Irini Haimala, Daphne N. Georgiadou, Diomi Mamma, Dimitris G. Hatzinikolaou

**Affiliations:** 1 Enzyme and Microbial Biotechnology Unit, Department of Biology, National and Kapodistrian University of Athens, 15772 Athens, Greece; agalanop@eie.gr (A.P.G.); irinihaimala@hotmail.com (I.H.); dgeorgiadou@biol.uoa.gr (D.N.G.); 2 Biotechnology Laboratory, School of Chemical Engineering, National Technical University of Athens, 15780 Athens, Greece

**Keywords:** xylan hydrolysis, *Byssochlamys spectabilis* ATHUM 8891 (*Paecilomyces variotii* ATHUM 8891), xylanase, β-xylosidase, acid stable enzymes, thermophilic enzymes

## Abstract

Two novel xylanolytic enzymes, a xylanase and a β-xylosidase, were simultaneously isolated and characterized from the extracellular medium of *Byssochlamys spectabilis* ATHUM 8891 (anamorph *Paecilomyces variotii* ATHUM 8891), grown on Brewer’s Spent Grain as a sole carbon source. They represent the first pair of characterized xylanolytic enzymes of the genus *Byssochlamys* and the first extensively characterized xylanolytic enzymes of the family *Thermoascaceae*. In contrast to other xylanolytic enzymes isolated from the same family, both enzymes are characterized by exceptional thermostability and stability at low pH values, in addition to activity optima at temperatures around 65 °C and acidic pH values. Applying nano-LC-ESI-MS/MS analysis of the purified SDS-PAGE bands, we sequenced fragments of both proteins. Based on sequence-comparison methods, both proteins appeared conserved within the genus *Byssochlamys*. Xylanase was classified within Glycoside Hydrolase family 11 (GH 11), while β-xylosidase in Glycoside Hydrolase family 3 (GH 3). The two enzymes showed a synergistic action against xylan by rapidly transforming almost 40% of birchwood xylan to xylose. The biochemical profile of both enzymes renders them an efficient set of biocatalysts for the hydrolysis of xylan in demanding biorefinery applications.

## 1. Introduction

A consortium of xylan-degrading enzymes, functioning at elevated temperatures and extreme pH values, is essential for high-yield lignocellullose hydrolysis to fuel 2nd-generation biorefinery applications [1,2]. Among them, endo-xylanases (EC 3.2.1.8) and β-xylosidases (EC 3.2.1.37) are two key hemicellulases that cleave the β-1,4 glycosidic linkage between two xylose units. Their action is differentiated by their preference towards either internal bonds in the xylan backbone and high MW xylo-oligosaccharides (xylanases) or the non-reducing termini of short chain-length xylooligosaccharides (β-xylosidases). Both enzymes find additional specific applications in the food, pulp and paper industry, either as constituents of enzymatic cocktails or as individual enzymes [3]. For all of the above reasons, numerous xylanases and β-xylosidases have been purified and characterized either from wild-type microbial strains or following overexpression in various hosts [4]. Fungal hemicellulases are among the most efficient and robust, in terms of stability and activity ranges [1,5], with most of them being characterized from efficient plant biomass degraders within the ascomycete and basidiomycete genera [2,6].

Of particular importance are the fungal lignocellulose degraders which, in addition to biomass hydrolysis, are capable of simultaneously fermenting the resulting monosaccharides for the production of bioethanol and/or other medium platform chemicals in a Consolidated Bioprocessing (CBP) approach [7]. This group of microorganisms are collectively referred to as CBP Type I strains and currently includes members from *Trichoderma*, *Fusarium*, *Aspergillus* and *Rhizopus* genera [8,9,10].

*Byssochlamys spectabilis* (anamorph of *Paecilomyces variotii*) is an ascomycete which, together with the genera *Paecilomyces* and *Thermoascus*, forms the *Thermoascaceae* family. Several *Thermoascaceae* members are often associated with food spoilage and human pathogenicity [11,12]. Various *B. spectabilis* strains have recently been recognized as producers of biotechnologically important enzymes and bioactive compounds [13,14,15,16]. *Byssochlamys spectabilis* ATHUM 8891, in particular, has been evaluated by our group as a potential Type I CPB strain showing promising traits, such as the production of biomass-degrading enzymes upon growth on various lignocellulosic substrates and fermentation of glucose and xylose to ethanol [17]. In that study, this particular *B. spectabilis* strain revealed a specific hemicellulose-tuned metabolism, since it presented higher ethanol yields on xylose fermentation compared to glucose, while in co-fermentation of glucose–xylose mixtures no distinguishable diauxic behavior was obtained.

In an attempt to acquire insight into the specific phenotype described above, we explored the xylanolytic arsenal of this strain. However, the available literature concerning the characterization of hemicellulases of *Byssohlamys* and the family *Thermoascaceae* in general is very limited [18,19], a fact that led us to seek and characterize the two key xylanolytic enzymes of the strain: PvXyn3A, which is a β-d-xylanase; and PvXyd11A, a β-1,4-d-xylosidase. The corresponding extracellular activities were produced in adequate quantities by the wild-type *B. spectabilis* ATHUM 8891. Consequently, both enzymes were purified from the supernatant of cultures in Brewer’s Spent Grain and biochemically characterized. Finally, the enzymes were subjected to nano-LC-ESI-MS/MS analysis in order to obtain information on their identity through the AA sequences of the corresponding fragments.

## 2. Materials and Methods

### 2.1. Strain and Growth Conditions

Enzymes were produced from the indigenous strain *Β. spectabilis* ATHUM 8891 [17]. Comparison of the 1670 bp 18S rDNA fragment of our strain (NCBI Accession# KU376436) against the nucleotide collection database of NCBI revealed as the closest phylogenetic neighbours, *B. spectabilis* strain IAM 13430 (AB023947), *P. variotii* strain UPSC 1651 (AF548080) and *B. spectabilis* CBS 101075 (AY526473) (100% identity with all). For the production of the two enzymes, the fungus was inoculated in four well-aerated 400 mL liquid cultures (1600 mL total culture volume), in 2 L Erlenmeyer flasks at 200 rpm and 25 °C. The basic medium used [17] was previously optimized for maximum xylanase production. Maximum extracellular activities were obtained after 7 days of growth at 50 g/L Brewers’ Spent Grain, pH 4, and growth temperature of 25 °C.

### 2.2. Enzyme Purification

Culture supernatants were concentrated through ammonium sulphate precipitation. The precipitate, received between 40 and 95% (NH_4_)_2_SO_4_ saturation levels, was equilibrated in 20 mM piperazine-HCl buffer, pH 5.5 with a PD-10 column (GE Healthcare, Chicago, IL, USA). The sample was applied into a custom-made Q-Sepharose-HR (GE Healthcare) anion exchange column (10 mL bed volume) equilibrated with the same buffer and eluted with a linear NaCl gradient from 0 to 500 mM in 20 mM piperazine-HCl, pH 5.5, at a flow rate of 1 mL/min. Xylanase and β-xylosidase were obtained as two distinct, but poorly separated, activity peaks between 200 and 300 mM NaCl. The corresponding fractions were pooled together, concentrated by lyophilization, and equilibrated in 20 mM citrate buffer pH 3.1 in a PD-10 column. The sample was placed into a custom-made 5 mL SP-Sepharose cation exchange column and eluted with a linear NaCl gradient (0 to 500 mM) at a flow rate of 0.6 mL min^−1^. A clear separation between the xylanase and β-xylosidase activities was achieved, since the two peaks eluted at 60 and 200 mM NaCl, respectively.

The two enzyme fractions were concentrated with Vivaspin2 ultrafiltration cartridges (Sartorius, Göttingen, Germany) of 3000 MW cut-off for xylanase and 10,000 MW cut-off for β-xylosidase. Each concentrated enzyme sample was further purified in a Sephacryl S-200 HR (GE Healthcare) gel filtration column (80 cm length × 1 cm diameter) eluted with a 50 mM citrate-phosphate buffer, pH 4, at a flow rate of 0.55 mL min^−1^. Pooled fractions of each enzyme activity were concentrated again in Vivaspin2 cartridges, filter sterilized (0.22 μm) and stored in sterile final elution buffer at 4 °C.

### 2.3. Enzyme Assays and Protein Concentration

Endo-β-1,4-xylanase (xylanase) activity was determined by incubating 50 μL of properly diluted enzyme sample with 450 μL of 2% (*w/v*) beechwood xylan (Sigma, St. Louis, MO, USA) in citrate-phosphate buffer (pH 4) in a thermoshaker (TS-100, BOECO, Hamburg, Germany) at 900 rpm and 50 °C. The reaction was stopped after 15 min by immediately placing samples into an ice bath and adding 500 μL of DNS reagent [20]. Residual xylan was removed by centrifugation (3 min at 12,000× *g*), the supernatant was transferred into a new tube, and boiled for 5 min. The concentration of reducing sugars was estimated by measuring the absorbance at 540 nm using a xylose standard curve.

The activity of β-xylosidase was estimated by incubating 10 to 50 μL of properly diluted enzyme samples in 1 mM p-Nitro-Phenyl-β-d-xylopyranoside (pNP-X) in 100 mM citrate-phosphate buffer, pH 4, at 1 mL final volume. Tubes were incubated for 10 min in a water bath at 50 °C. The reaction was immediately stopped by placing the tubes in an ice bath and adding 1 mL of 1 M NaCO_3_. Quantification of product accumulation was performed at 410 nm using a pNP standard curve prepared at the specific assay conditions. The blanks for both activity measurements were enzyme samples incubated at 121 °C for 15 min.

Enzyme activity was expressed in nkatals (nkat), defined as the amount of enzyme required for the release of one nmole equivalent xylose (xylanase) of pNP (β-xylosidase) per second under the conditions described above.

At all steps, protein concentrations were determined using the Bradford assay [21] and Bovine Serum Albumin (Sigma-Aldrich, St. Louis, MO, USA) as standard.

### 2.4. Biochemical Characterization

The effect of temperature on enzyme activity was determined by performing the regular enzyme assay at pH 4 for a temperature range from 30 to 80 °C. The reaction time was 5 min in order to minimize possible enzyme deactivation effects. pH optima were determined in a similar manner at 50 °C and 100 mM citrate-phosphate or phosphate buffers at a pH-range from 2.5 to 9. Temperature stability was studied by performing the standard enzyme assay using samples incubated at the indicated temperatures and pH 4 and for various time intervals. pH stability was determined in a similar manner, by incubating samples at 40 °C in 200 mM citrate-phosphate or phosphate buffers of different pH values. To avoid the pH of enzyme samples to alter the examined pH values, samples were diluted at least 30 times in the incubation buffer.

K_M_s were determined by measuring the initial reaction rates for initial xylan concentrations ranging from 0.5 to 10 g L^−1^ (xylanase) and initial pNP-X concentrations ranging from 0.1 to 20 mM (β-xylosidase).

Inhibition of Xyn3A by xylose was investigated by repeating the above set of initial reaction rate experiments, three additional times with initial xylose concentrations 5 mM, 20 mM and 50 mM. K_I_ value was determined by the corresponding Lineweaver–Burk plots applying Michaelis–Menten analysis for competitive inhibition.

The effect of various chemicals (modulators) on the activity of both enzymes was determined under the regular assay conditions with the addition of the indicated concentrations of each compound in the final assay mixture.

Reduced MW determinations and sample purity levels were estimated by SDS-PAGE and Coomassie staining. The non-reduced MW of the enzymes was calculated from their elution volume in a Sephacryl S-200 (GE-Healthcare) gel filtration column set-up (1 m length, 1 cm internal diameter). For the calibration of the column, cytochrome C (12.4 kDa), peroxidase (40 kDa), bovine serum albumin (66.5 kDa), glucose oxidase (150 kDa) and catalase (232 kDa) standards were used. Samples and standard proteins were injected into the column and MW’s were determined from the log(MW) vs. elution volume plots through linear regression.

Zymogram analysis for xylanase activity was performed under denaturing conditions in a 12% SDS-PAGE gel containing 0.5% *w/v* beechwood xylan. Following electrophoresis, the gel was washed for 30 min in 1% Triton X-100 solution at room temperature and then incubated for 30 min at 50 °C in 100 mM citrate-phosphate buffer pH 4. Following several washings with deionized water, the gel was stained with 1 mg/mL Congo Red solution for 2 h at room temperature and destained with 1 Μ NaCl.

The carbohydrate content of the purified enzyme preparations was determined by the phenol-sulfuric acid method [22]. In addition, it is expressed as % (*w/w*) equivalent mannose (standard).

Isoelectric points were determined through chromatofocusing in PBE 94 column using Polybuffer 74 at a pH-range from 6 to 4, according to the manufacturer’s recommendations.

### 2.5. Hydrolysis Experiments

Xylan hydrolysis experiments were performed using beechwood xylan and the purified sample preparations. Either 25 nkat PvXyn11A or 25 nkat PvXyn11A plus 2.5 nkat PvXyd3A, were added in 900 μL of xylan suspension (25 mg mL^−1^) in 50 mM citrate-phosphate buffer, pH 4. All reaction tubes were incubated in a thermoshaker at 55 °C and 900 rpm. The same reaction mixtures without enzyme addition were used as blanks. 100 μL samples were withdrawn at specific time intervals, filtered (0.22 μm) and analyzed for X1 to X4 xylooligosaccharides through HPLC. The analysis was performed in an HP5100 system (Hewlett-Packard, Palo, CA, USA), using two Agilent Hi-Plex-Pb columns connected in series. The columns were maintained at 70 °C and eluted with HPLC-grade water at a flowrate of 0.6 mL min^−1^. Detection was performed using an RI detector (HP5500).

### 2.6. Nano LC ESI-MS/MS Analysis and Protein Identification

**Figure 1:**
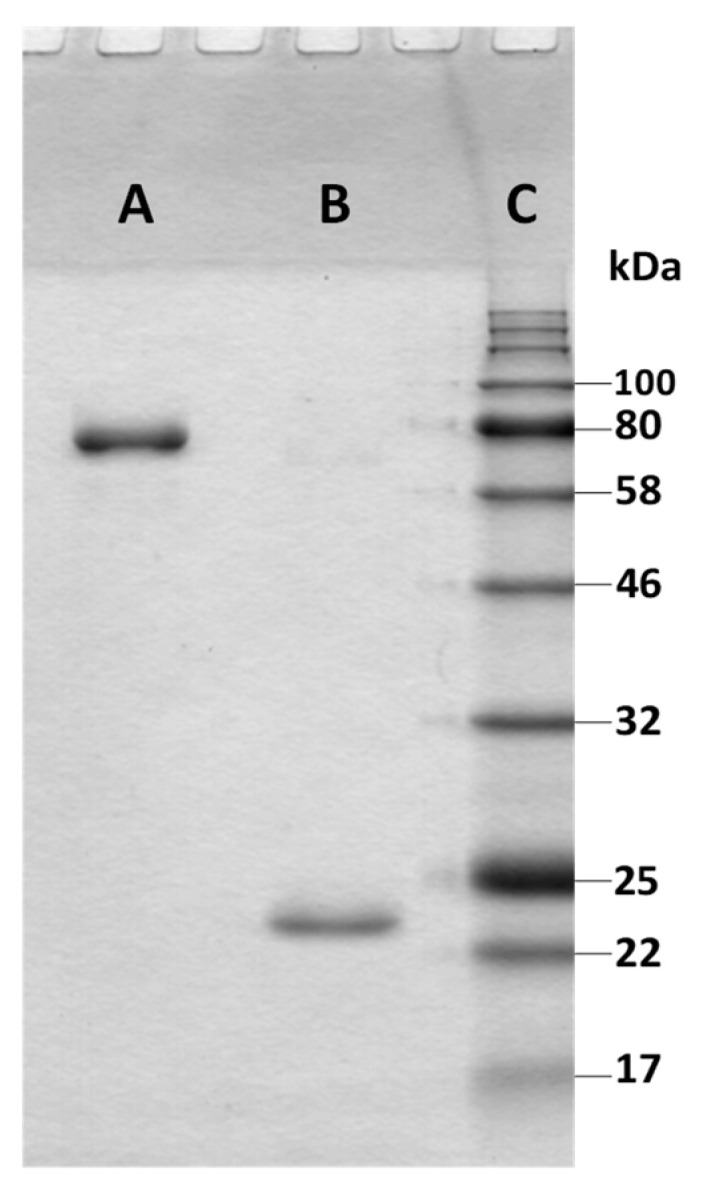
SDS-PAGE of the purified *B. spectabilis* ATHUM 8891 hemicellulases. (**A**) PvXyd3A, (**B**) PvXyn11A, (**C**) Protein marker NEB (#P7712).

The single band from Coomassie stained SDS gel of both xylanase and β-xylosidase (Figure 1) was excised, cut into smaller pieces and subjected to in-gel tryptic digestion according to established protocols [23]. The obtained peptides were subjected to nanoLC separation using an UltiMate3000 nanoRSLC system (ThermoFisher Scientific, Germering, Germany) operated in a trap column setup [23] applying a 25 cm separation column (C18, 100 Å, 3 µm bead size, 75 µm inner diameter, 25 cm length; ThermoFisher Scientific) and a linear 120 min gradient. The nanoLC effluent was continuously analyzed by an online-coupled electrospray ionization (Captive spray; Bruker Daltonik GmbH, Bremen, Germany) ion-trap mass spectrometer (amaZon speed ETD; Bruker Daltonik GmbH) with 20 MS/MS acquired per full scan MS applying precursor exclusion for 0.2 min. Three independent SDS-PAGE separated bands were analyzed for xylanase and xylosidase, respectively.

Proteins were annotated by performing local Blast against the available genomes and proteomes of the family *Thermoascaceae* retrieved from the GenBank assembly database [24] as well as the characterized sequences of the dbCAN database (release 8, last update 8 August 2019) [25].

### 2.7. Statistical Analysis

All experiments were conducted at least in triplicate, with error bars representing the standard deviation among measurements. SigmaStat and SigmaPlot software were used for statistical analysis (ANOVA) and data presentation, respectively.

## 3. Results

### 3.1. Enzyme Production and Purification

The production of xylanolytic enzymes from *B. spectabilis* ATHUM 8891 is induced by the presence of lignocellulosic substrates in the medium [17]. Thus, we used Brewer’s Spent Grain as a carbon source for the induction of the two hemicellulases. For the purification of the two enzymes, we used culture supernatants upon seven days of growth at optimum conditions. At this time point, the supernatant xylanase and β-xylosidase activities were 148 and 7.9 nkat/mL, respectively, allowing the initiation of the purification steps. The steps we followed for the purification of the two enzymes are summarized in Table 1.

Initially we performed ammonium sulfate precipitation from the culture supernatant followed by anion exchange chromatography on Q-Sepharose. Those two steps led to 3.1 times enrichment and 76% recovery for PvXyn11A and 7.5 times enrichment and 84% recovery for PvXyd3A (Figure S1). To further purify the two enzymes, we employed cation exchange chromatography using an SP Sepharose column where, at pH 3.1, we obtained the complete separation of the two enzymes (Figure S2). The last purification step was gel filtration, which yielded specific activities of 75,800 nkat/mL for PvXyn11A and 5200 nkat/mL for PvXyd3A. Overall, the purification process led to electrophoretically pure enzyme solutions (Figure 1) and over 50% recoveries for both enzymes (Table 1).

Zymogram analysis using xylan as substrate revealed a clear halo of xylan hydrolysis at the anticipated MW for PvXyn11A, in both the crude extracellular extract as well as in the purified xylanase sample (Figure S3). As expected, no halo was observed in the purified β-xylosidase lane.

### 3.2. Activity and Stability Properties

**Figure 2:**
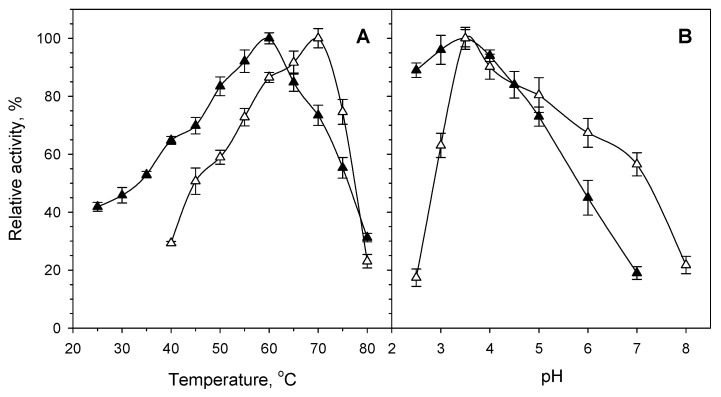
Effect of temperature ((**A**) determined at pH 4) and pH ((**B**) determined at 50 °C) on the activity of the purified PvXyn11A (▲) and PvXyd3A (△) of *B. spectabilis* ATHUM 8891.

**Figure 3:**
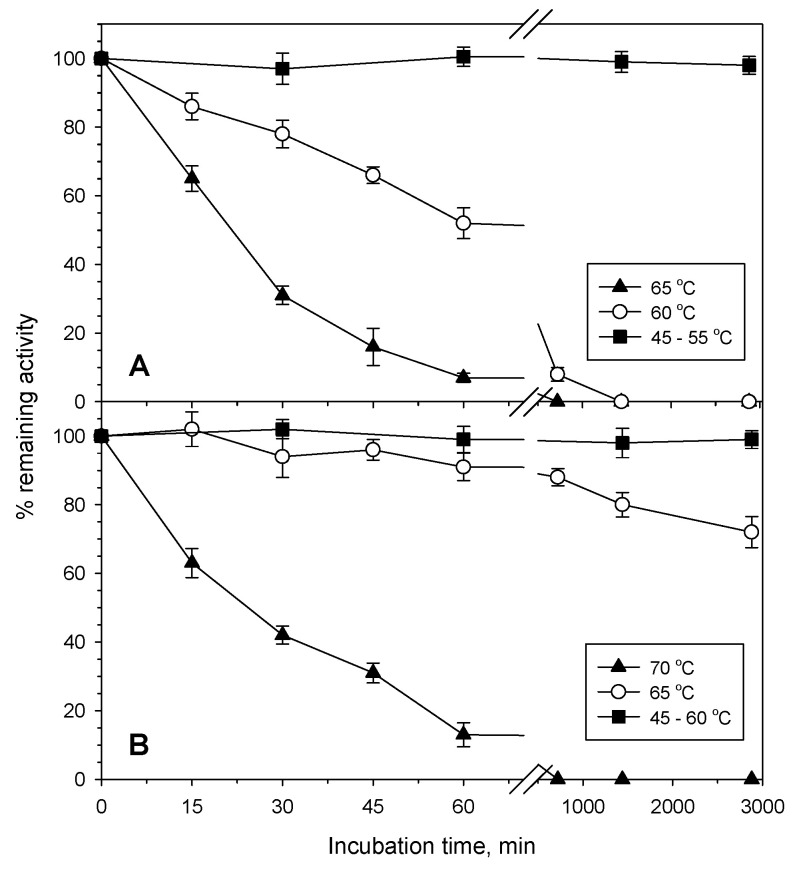
Thermal stability of PvXyn11A (**A**) and PvXyd3A (**B**) purified from *B. spectabilis* ATHUM8891 determined in citrate-phosphate buffer pH 4.

In accordance with the thermophilic nature of the genus *Byssochlamys*, PvXyn11A and PvXyd3A revealed thermophilic characteristics. PvXyn11A had a temperature optimum at 60 °C, exhibiting over 70% of its maximum activity within a temperature range between 45 °C and 70 °C (Figure 2A). PvXyn11A also appeared very stable up to 55 °C, where it retained its initial activity for at least 24 h of incubation. At 60 °C and 65 °C, PvXyn11A retained 50% of its initial activity upon 45 min and 20 min of incubation, respectively (Figure 3A). Regarding PvXyd3A, the temperature optimum was even higher, at 70 °C. Beyond this temperature, though, activity dropped rapidly, probably due to the instability of the enzyme at these temperatures. PvXyd3A revealed higher thermostability compared to the xylanase as it retained its initial stability for at least 24 h at 60 °C. At 70 °C, though, the activity of PvXyd3A decreased to the 50% of the initial from the first 20 min of incubation (Figure 3B).

**Figure 4:**
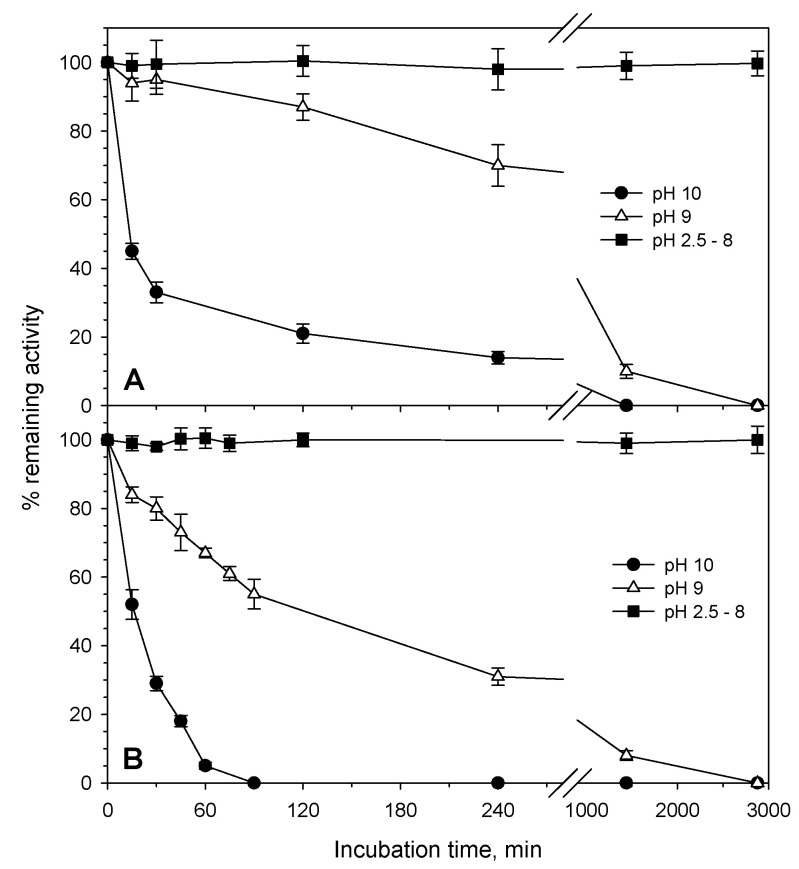
pH stability analysis for the purified PvXyn11A (**A**) and PvXyd3A (**B**) of *B. spectabilis* ATHUM 8891 at 40 °C.

Both enzymes revealed a preference for acidic environments. PvXyn11A exhibited its highest activity at pH 3.5. The activity of PvXyn11A dropped gradually at higher pH values, while in values as low as 2.5, the activity remained at approximately 90% of the optimum (Figure 2B). The enzyme was stable upon incubation within a broad pH range from 2.5 to 8, retaining 100% of its activity for at least 48 h (Figure 4). Similarly, PvXyd3A had an optimum pH at 3.5, while it retained more than 50% of its optimum activity within the pH range 3 to 7 (Figure 2B). Similar to the xylanase, the activity of PvXyd3A was not affected by the prolonged incubation in buffers with pH from 2.5 to 8 (Figure 4).

The effect of a variety of metal ions, detergents and reducing agents on the xylanolytic efficiency of PvXyn11A and PvXyd3A was also determined. The majority of the metal ions examined did not cause any significant impact on the activity of both enzymes since the observed activities did not decrease more than 35% for metal concentrations up to 10 mM (Table 2).

However, at the highest concentrations tested, Hg^2+^ and Cu^2+^ inhibited PvXyn11A activity by almost 100% and 50%, respectively. Among the detergents and reducing agents tested, only dithiotreitol (DTT) had an 50% inhibitory effect on the activity of PvXyd3A at 10 mM.

### 3.3. Substrate Specificity and Action on Xylan

PvXyn11A exhibited the same levels of specific activity for birchwood and beechwood xylan (approx. 76,000 nkat/mg protein), while the specific activity for oat-spelt xylan was 27% lower. Activity levels less than 3% compared to those on xylan were observed when using carboxy-methyl cellulose (CMC), phosphoric acid swollen cellulose (PASC), pNP-β-D-xylopyranoside (pNP-X) and pΝP-β-D-glucopyranoside (pNP-G) as substrates. The K_M_ of the purified PvXyn11A for beechwood xylan was found to be equal to 2.52 ± 0.45 g/L (Table 3).

PvXyd3A was highly active against the xylobiose analogue, pNP-X, with a K_M_ of 0.48 ± 0.06 mM (Table 4). In contrast, we only managed to detect traces of xylanolytic activity when we incubated PvXyd3A together with the three different types of xylan: birchwood, beechwood and oat-spelt xylan. Xylose, the product of xylobiose hydrolysis, was found to competitively inhibit the β-xylosidase activity of PvXyd3A with K_I_ equal to 10.2 mM (data not shown).

**Figure 5:**
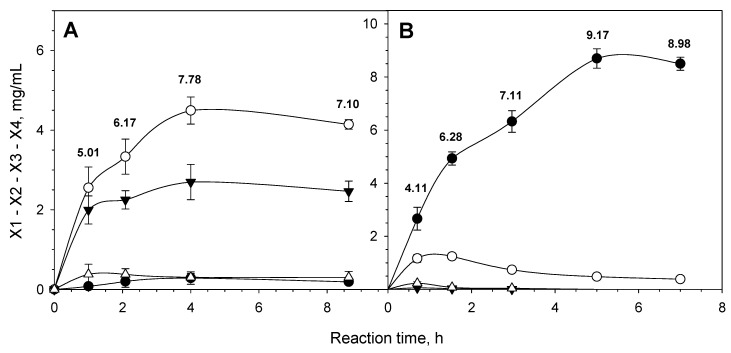
Hydrolysis of beechwood xylan (initial concentration of 25 g/L) by only the xylanase PvXyn11A (25 nkat/mL—(**A**)) and a mixture of PvXyn11A and PvXyd3A (25 nkat/m–2.5 nkat/mL—(**B**)) at pH 4 and 50 °C. ●, xylose (X1); ○, xylobiose (X2); ▼, xylotriose (X3); ∆, xylotetraose (X4). Numbers at each time point indicate the sum of X1 to X4 (mg/mL). Data are the means of triplicate reactions with standard deviations.

When beechwood xylan was hydrolyzed by PvXyn11A, the main reaction products were xylobiose and xylotriose at a final analogy of 5/3 (Figure 5A). Almost 7.8 g/L of X1 to X4 xylooligosaccharides were produced at the end of the reaction, representing xylan conversion of over 30%. This result indicates that PvXyn11A is a true endo-xylanase, and it is able to efficiently hydrolyze xylooligosaccharides constituted by four or more xylose units.

Addition of PvXyd3A to the hydrolysis reaction yielded xylose as the main reaction product (>90%). In the latter case, the reaction reached a plateau almost an hour later, with the total amount of the produced xylo-oligosaccharides (X1 to X4) being 20% higher (Figure 5B), corresponding to xylan to xylose conversion of almost 40%. The increase in the total xylooligosaccharides produced upon PvXyn3A addition implies a significant inhibition of PvXyn11A by xylobiose, an effect that is alleviated by the addition of PvXyd3A.

### 3.4. Sequence Determination: Taxonomic Distribution and CAZy Classification

The sequenced fragments retrieved from the Nano-LC- ESI-MS/MS analysis were searched in the two available proteomes and the nine genomes of the family *Thermoascaceae* (Supplementary Tables S1 and S2). Our analysis indicates that both enzymes are conserved within the genus *Byssochlamys*, since hits for the fragments of both enzymes were found in the seven out of the eight *Byssochlamys* sp. with available genomic data. Additionally, homologue fragments of PvXyn11A and PvXyd3A were also found in the two other sequenced genomes of *Thermoascaceae* members, indicating that the isolated enzymes of *B. spectabilis* ATHUM 8891 are conserved not only within the genus *Byssochlamys*, but also within the entire *Thermoascae* family.

The two enzymes were further annotated according to CAZy database; based on sequence similarities with CAZY entries, the Xyn11A fragment belongs to the GH11 domain. The closest characterized entries possess xylanolytic activity and are encountered in the genus *Phanerochaete chrysosporium*. The Xyd3A fragments were classified within Glycoside Hydrolase family 3. The closest characterized entries are β-xylosidases, and they belong to the genera Rasamonia, Aspergillus, Talaromyces and Neurospora. However, there were not available biochemical characteristics for those CAZY entries besides their substrate specificity (E.C number) against xylan.

## 4. Discussion

Although a potential role in ethanol production from lignocellulose has been identified for *Thermoascaceae* members [36], the majority of the studies in this area have mainly been focused on other fungal genera such as *Fusarium* and *Neurospora* spp. However, the hemicellulose-tuned metabolism of *B. spectabilis* ATHUM 8891 [17] indicates an intriguing hydrolytic potential, prompting us to examine the major xylan degrading enzymes of the strain. Thus, in the present work, we report the purification and characterization of a β-1,4-xylanase, PvXyn11A, and a β-xylosidase, PvXyd3A, which can be efficiently secreted and isolated from the culture supernatant of the fungus upon growth on hemicellulose-rich substrates.

The specific activity achieved for PvXyn11A was by far the highest reported by other *Thermoascaceae* strains and the second highest when compared with heterologously expressed xylanases of the family in other expression systems (Table 3). The obtained specific activity for PvXyd3A was also the highest reported among β-xylosidases isolated from *Thermoascaceae* members (Table 4), as well as being among the highest observed for fungal β-xylosidases in general [37]. Higher specific activity values have only been reported for the wild-type enzymes of *Aspergillus niger* GS1 [38] and *Penicillium janczewskii* CRM 1348 [39].

The thermophilic nature of PvXyn11A is in line with the thermal properties of other xylanases of the genus *Byssochlamys* (*Paecilomyces*), while it tolerates substantially lower temperatures than xylanases of other *Thermoascaceae* genera (Table 3). Intriguingly, PvXyd3A stands out for its thermal properties; the enzyme was demonstrated to be the most thermophilic among *Thermoascaceae*-derived β-xylosidases (Table 4) and was superior to most fungal and also to several bacterial thermophilic β-xylosidases [37]. To our knowledge, similar thermal characteristics have only been reported for three other GH3 β-xylosidases: those isolated from *Aspergillus japonicus* MU-2 [40], *Aspergillus niger* GS1 [38] and *Aspergillus ochraceus* [41].

Compared to the other hemicellulases isolated from members of the family *Thermoascaceae* (Table 3 and Table 4), the enzymes of *B. spectabilis* ATHUM 8891 proved the most acidophilic. The xylanase and β-xylosidase of the phylogenetically closest *P. variotii* IMD RK 032 had similar pH-optima (pH 4) without any stability data available [18]. Although fungal hemicellulases have neutral to acidic pH optima, an optimum activity below 4 is rarely encountered. We were able to find only two xylanases with pH optima in this range, namely the xylanase I of *Penicillium sclerotium*, with a reported pH-activity optimum of 2.5 [42], and the xylanase from the basidiomycete *Laetiporus sulphureus*, with a pH optimum of 3 [43]. In addition, the acidophilic GH10 xylanase of *Bispora* sp. MEY-1, upon expression in *P. pastoris*, revealed similar pH stability properties, but the optimum pH for the activity of this enzyme was 5 [44].

PvXyd3A with a pH optimum at 3.5 is also among the most acidophilic fungal β-xylosidases. Similar highly acidic pH optima have been reported for the β-xylosidases of *Aspergillus pulverulentus* (2.5–3.5) [45], *Aureobasidium* sp. (3) [46], *Aureobasidium pullulans* (3.5) [47] and *Penicillium sclerotium* (2.5) [48]. All these β-xylosidases are also quite stable at low pH values (<4), like PvXyd3A. At this pH range, significant stability is also observed for several β-xylosidases from *Aspergillus* species that have higher pH optima, such as the β-xylosidase of *A. japonicus* MU-2 [40].

Among the examined effectors, DDT had an inhibitory effect on the activity of PvXyd3A only, indicating the importance of disulfide bonds for the correct folding and function of the enzyme. SDS and EDTA effects on activity, on the other hand, were marginal for both enzymes, indicating a minor role for hydrophobic interactions and metal ions in the maintenance of the three-dimensional structure of the proteins.

The K_M_ found for PvXyn11A is among the lowest reported among the genus *Thermoascace* and in accordance with those reported for other *P. variotii* strains (Table 3). On the other hand, though, the PvXyd3A K_M_ is an order of magnitude lower than the second lowest value reported for xylosidases of the *Thermoascaceae* family (Table 4), indicating a much higher affinity for this substrate. K_M_ values for pNP-X of around 0.5 mM, as reported for PvXyd3, are common for the β-xylosidases from several other known xylanolytic fungal species [40,41].

Sequence comparisons of the retrieved sequenced fragments within the available genomic data of the genus *Paecilomyces* showed that both enzymes are conserved not only within the genus *Byssochlamys*, but throughout the *Thermoascaceae* family. Additional searches against the characterized entries of the CAZy database classified PvXyd3A as a member of the Glycoside Hydrolase Family 3 and PvXyd11A a member of the Glycoside Family 11, with the closest characterized members belonging to different fungal families.

These two novel enzymes represent a thermostable and highly acidophilic set of biocatalysts for the efficient hydrolysis of xylan in demanding biorefinery applications. Their unique properties seem to play a central role in defining the unique hemicellulose-tuned character of *B. spectabilis* ATHUM 8891, which we aim to further elucidate through its full-genome analysis and further studies on the physiology of the strain.

## Figures and Tables

**Table 1:** Summary table of the steps followed for the purification of PvXyn11A and PvXyd3A.

Purification Step	Xylanase			β-Xylosidase		
	Specific Activity (nkat mg^−1^)	Enrichment	Recovery (%)	Specific Activity (nkat mg^−1^)	Enrichment	Recovery (%)
Extracellular medium	1259	1.0	100	67	1	100
(NH_4_)_2_SO_4_ precipitation	1418	1.1	92	89	1.3	88
Q-Sepharose anion exchange	3957	3.1	76	504	7.5	84
SP-Sepharose cation exchange	29,781	23.7	72	3359	50.1	62
Sephacryl S-200 gel filtration	75,847	60.2	62	5200	77.6	51

**Table 2:** Effect of various modulators on the relative activity (%) of PvXyn11A and PvXyd3A from *B. spectabilis* ATHUM 8891.

Compound	PvXyn11A		PvXyd3A	
	1 mM	10 mM	1 mM	10 mM
**Control**	100	100	100	100
**CuCl_2_**	60 ± 5	39 ± 4	95 ± 3	92 ± 3
**CoCl_2_**	90 ± 6	78 ± 5	105 ± 7	97 ± 6
**NaCl**	102 ± 4	109 ± 8	95 ± 6	95 ± 9
**BaCl_2_**	98 ± 5	103 ± 2	88 ± 3	86 ± 5
**CaCl_2_**	107 ± 4	110 ± 7	95 ± 7	92 ± 5
**HgCl_2_**	86 ± 6	7 ± 3	95 ± 3	66 ± 7
**MgSO_4_**	90 ± 7	93 ± 8	103 ± 6	99 ± 3
**Pb(CH_3_COO)_2_**	106 ± 7	95 ± 5	106 ± 3	99 ± 4
**MnSO_4_**	94 ± 5	88 ± 6	104 ± 5	138 ± 5
**ZnSO_4_**	88 ± 10	85 ± 7	106 ± 9	122 ± 6
**SDS**	84 ± 5	80 ± 2	81 ± 3	72 ± 5
**EDTA**	94 ± 5	90 ± 4	99 ± 2	95 ± 3
**DDT**	98 ± 6	103 ± 7	81 ± 4	50 ± 7

**Table 3:** Biochemical properties of the characterized xylanases originated from *Thermoascaceae* members.

	*P. thermophila* J18	*P. thermophila* J18	*P. thermophila* J18	*Thermoascus crustaceus* JCM12803	*Thermoascus auranticus*	*P. variotii*	*P. variotii* IMD RK 032	*B. spectabilis* ATHUM8891
**Production Source**	WT	*E. coli*	*P. Pastoris*	*P. Pastoris*	WT	WT	WT	WT
**MW**	26 kDa	28 kDa	29 kDa	ND	32	25 kDa	20 kDa	23 kDa
**pI**	ND	4.43	ND	ND	7.1	3.9	5.2	>3 & <4
**C(H_2_O) content**	21%	ND	ND	ND	ND	4.5%	No glycosylation	7.1%
**Specific activity**	20,500 nkat/mg (BiX)	11,800 nkat/mg (BiX)	108,950 nkat/mg (BiX)	24,716 nkat/mg (BeX)	1064 nkat/mg (OAX)	8200 (LX)	816 nkat/mg (xylan)	75,800 nkat/mg (BeX)
**T optimum**	75 °C	75 °C	75 °C	65–70 °C	80 °C	65 °C	50 °C	60 °C
**T stability**	Stable up to 75 °C for 30 min	t_1/2_ 174.8, 137.9, 107.4 and 68.2 min at 70, 75, 80 and 85 °C, resp.	Stable up to 70 °C for 30 min. 75% residual act. at 75 °C and 65% at 80 °C (30 min)	Stable for 60 min at least at 60 °C	60 min at 80 °C. Stable up to 70 °C for days	Stable up to 60 °C. 65% residual activity after 60 min at 70 °C, inactivation after 40 min at 80 °C	ND	t_1/2_ of 17 and 60 min at 65 and 60 °C. Stable up to 55 °C for 2 days at least
**pH optimum**	6.5	7	7	5	5	5.5–7	4	3.5
**pH stability**	Stable for 30 min at least at pH 6.0–11.0	Stable for 30 min at least at pH 6.5–10.5	Stable for 30 min at least at pH 4.5–11	Stable at pH 3–11	ND	Stable at pH 3–10	ND	Stable for 2 days at least at pH 2.5–8
**K_M_**	ND	4.4 (BiX), 3.6 mg/mL (BeX), 9.7 mg/mL (OAX)	ND	ND	1.7 mg/mL (OAX)	2.5 mg/mL (LX)	49.5 mg/mL (xylan)	2.52 ± 0.45 mg/mL (BeX)
**GH family**	ND	11	ND	ND	ND	ND	ND	11
**Ref.**	[26]	[27]	[28]	[29]	[30]	[19,31]	[18]	Present work

**Table 4:** Biochemical properties of the characterized β-xylosidases originated from *Thermoascaceae* members.

	*P. thermophila* J18	*P. thermophila* J18	*P. thermophila* J18	*Thermosascus* sp.	*P. variotii* IMD RK 032	*B. spectabilis* ATHUM 8891
**Production source**	WT	*E. coli*	*P. pastoris*	WT	WT	WT
**MW**	53.5 kDa	52. kDa	52.3 kDa	100 kDa	67 kDa	78 kDa
**pI**	ND	ND	ND	ND	4	>3 & <4
**Specific activity**	724 nkat/mg (pNPX)	765 nkat/mg (pNPX)	4.4 nkat/mg (pNPX)	116.7 nkat/mg (pNPX)	ND	5200 nkat/mg (pNPX)
**T optimum**	55 °C	55 °C	60 °C	55 °C	60 °C	70 °C
**T stability**	Stable up to 55 °C for 30 min	t_1/2_ of 1160, 605 and 15 min at 50, 55 and 60 °C	ND	Stable up to 60 °C for 1 h.	ND	t_1/2_ of 25 min at 70. Stable up to 60 °C for 2 days at least
**pH optimum**	6.5	7	7	4.5	4	3.5
**pH stability**	Stable 30 min from 6 to 9 at 50 °C	Stable 30 min from 6 to 9 at 50 °C	ND	ND	ND	Stable for 2 days at least at pH 2.5–8
**K_M_ (pnpX)**	4.3 mM	4.5 mM	8 mM	ND	5.4 mM	0.45 ± 0.06 mM
**K_I_ (xylose)**	139 mM	ND	ND	ND	ND	10.2 mM
**GH**	ND	GH43	ND	ND	ND	3
**Ref.**	[32]	[33]	[34]	[35]	[18]	Present work

## Data Availability

The data that support the findings of this study are available from the corresponding author upon reasonable request.

## Supplementary Materials

The following are available online at https://www.mdpi.com/article/10.3390/jof7060430/s1, Figure S1: Anion exchange chromatography on Q-Sepharose, for the purification of PvXyn11A and PvXyd3A from B. spectabilis ATHUM 8891. The buffer system used was piperazine-HCl, at pH 5.5. Specific conditions are described in Materials and Methods, Figure S2: Cation exchange chromatography on SP-Sepharose, for the purification of for the purification of PvXyn11A and PvXyd3A from *B. spectabilis* ATHUM 8891. The buffer system used was citrate-NaOH, at pH 3.1. Specific conditions are described in Materials and Methods, Figure S3: Zymogram of *B. spectabilis* ATHUM 8891 hemicellulases in a 12% SDS-PAGE gel containing 0.5 *w/v* beechwood xylan (see Materials and Methods). A and B, NEB protein standard P7712S before and after Congo Red treatment, respectively; C, crude extracellular extract; D, purified xylanase standard; E, purified β-xylosidase sample, Table S1: Complete LS-MS/MS data of the Xyd3A and Xyn11A fragments. Proteins are identified in *Thermoascaceae* genomes using local tblastn and an e value cut-off of 0.001, Table S2: Complete LS-MS/MS data of Xyd3A and Xyn11A fragments. Proteins are identified in *Thermoascaceae* proteomes using local blastp for oligopeptides and an e value cut-off of 0.001. For every individual search, only the hits with the best scores are shown.
