# Supplementary material for: Characterization of the Highly Efficient Acid-Stable Xylanase and β-Xylosidase System from the Fungus Byssochlamys spectabilis ATHUM 8891 (Paecilomyces variotii ATHUM 8891)
Source: J Fungi (Basel). 2021 May 29;7(6):430. doi: 10.3390/jof7060430 (PMC8228849; doi:10.3390/jof7060430)
Supplement: Supplementary file 1 [file jof-07-00430-s001.zip › Figures.pdf]

Figure S1. Anion exchange chromatography on Q-Sepharose, for the purification of PvXyn11A and PvXyd3A from *B. spectabilis* ATHUM 8891. The buffer system used was piperazine-HCl, at pH 5.5. Specific conditions are described in Materials and Methods.

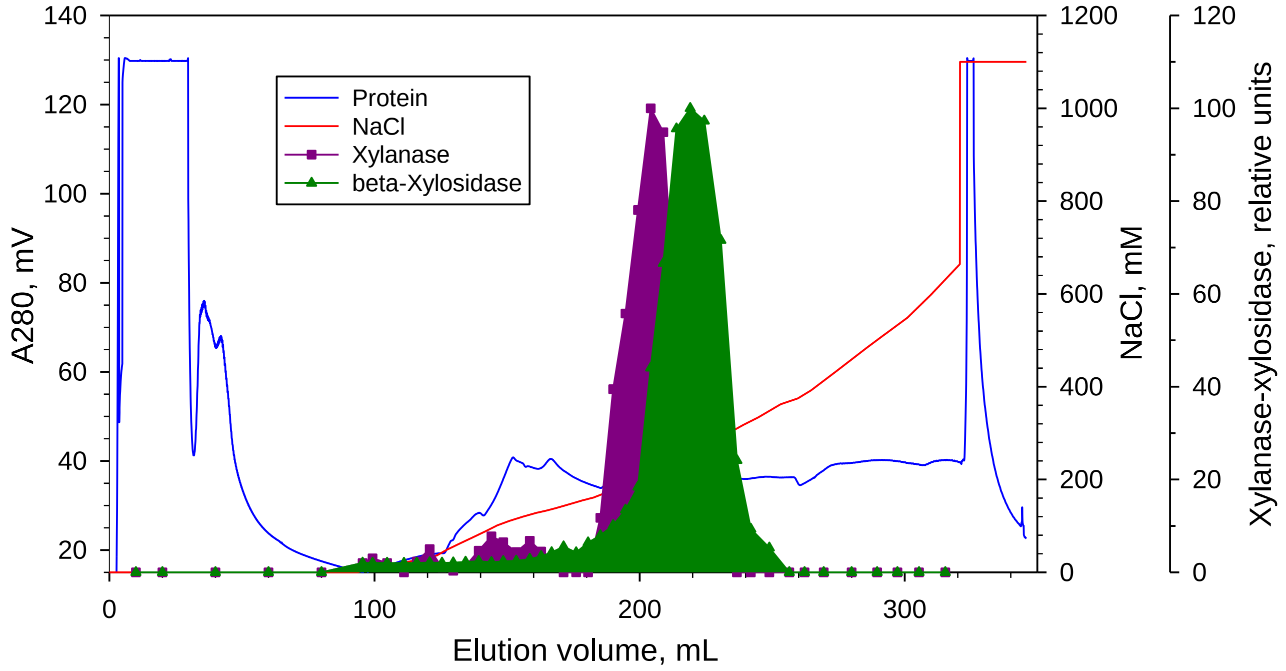

Figure S2. Cation exchange chromatography on SP-Sepharose, for the purification of for the purification of PvXyn11A and PvXyd3A from *B. spectabilis* ATHUM 8891. The buffer system used was citrate-NaOH, at pH 3.1. Specific conditions are described in Materials and Methods.

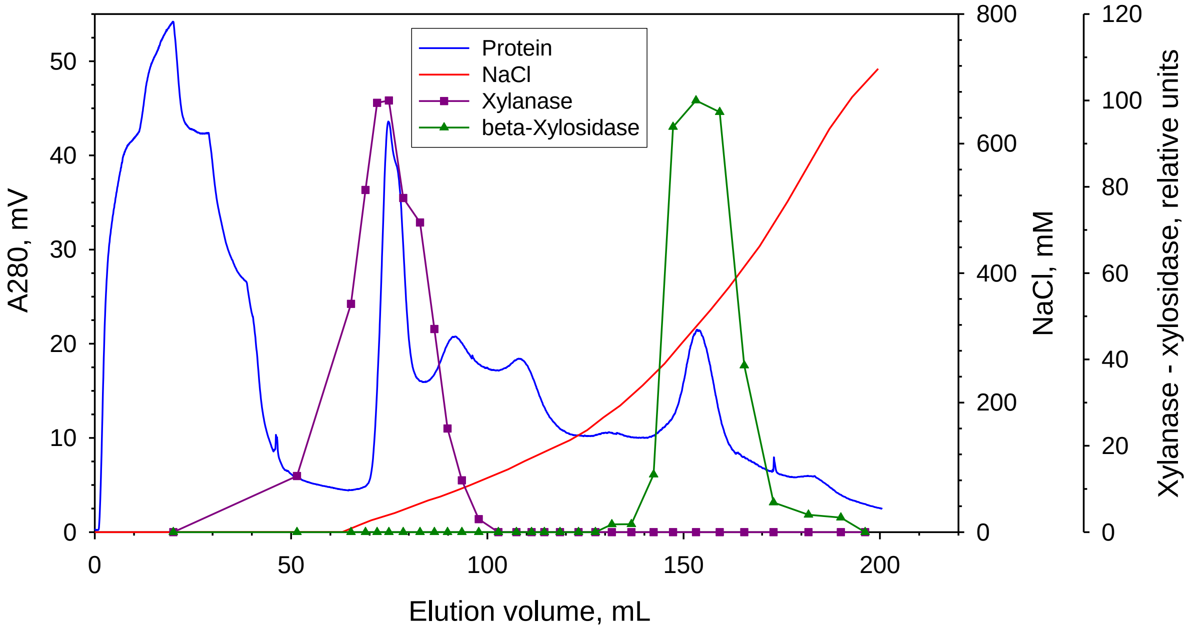

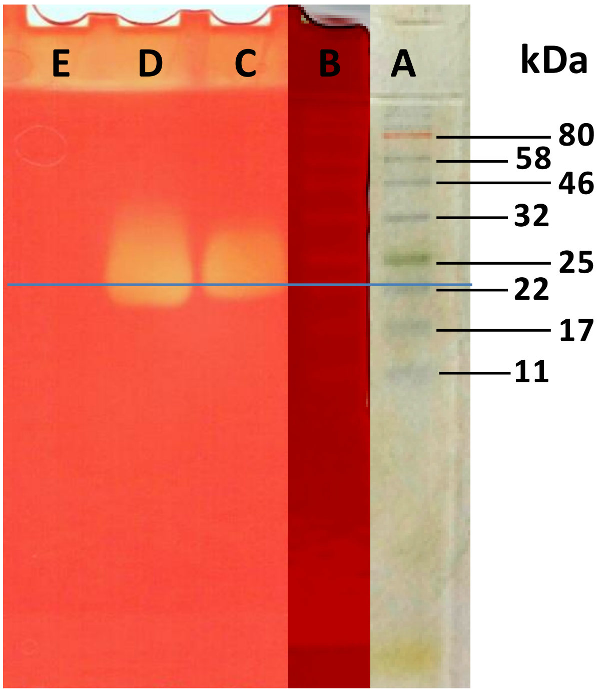

Figure S3. Zymogram of *B. spectabilis* ATHUM8891 hemicellulases in a 12% SDS-PAGE gel containing 0.5 w/v beechwood xylan (see Materials and Methods). A and B, NEB protein standard P7712S before and after Congo Red treatment, respectively; C, crude extracellular extract; D, purified xylanase standard; E, purified β-xylosidase sample.
